# *Streptococcus mutans* extracellular DNA levels depend on the number of bacteria in a biofilm

**DOI:** 10.1038/s41598-018-31275-y

**Published:** 2018-09-06

**Authors:** Miah Kim, Jaegyu Jeon, Jaegon Kim

**Affiliations:** 1 Department of Conservative Dentistry, Chonbuk National University, 567 Baekjaedaero, Jeonju-city Jeonbuk, 54896 South Korea; 2 Department of Preventive Dentistry, School of Dentistry, Chonbuk National University, 567 Baekjaedaero, Jeonju-city Jeonbuk, 54896 South Korea; 3 Department of Pediatric Dentistry, School of Dentistry, Chonbuk National University, 567 Baekjaedaero, Jeonju-city Jeonbuk, 54896 South Korea

## Abstract

*Streptococcus mutans* is a component of oral plaque biofilm that accumulates on the surface of teeth. The biofilm consists of extracellular components including extracellular DNA (eDNA). This study was conducted to investigate the factors that may affect the eDNA levels of *S. mutans* in biofilms. For the study, *S. mutans* UA159 biofilms were formed for 52 h on hydroxyapatite (HA) discs in 0% (w/v) sucrose +0% glucose, 0.5% sucrose, 1% sucrose, 0.5% glucose, 1% glucose, or 0.5% sucrose +0.5% glucose. Acidogenicity of *S. mutans* in the biofilms was measured after biofilm formation (22 h) up to 52 h. eDNA was collected after 52 h biofilm formation and measured using DNA binding fluorescent dye, SYBR Green I. Biofilms cultured in 0.5% sucrose or glucose had more eDNA and colony forming units (CFUs) and less exopolysaccharides (EPSs) than the biofilms cultured in 1% sucrose or glucose at 52 h, respectively. The biofilms formed in 0% sucrose +0% glucose maintained pH around 7, while the biofilms grown in 0.5% sucrose had more acidogenicity than those grown in 1% sucrose, and the same pattern was shown in glucose. In conclusion, the results of this study show that the number of *S. mutans* in biofilms affects the concentrations of eDNA as well as the acidogenicity of *S. mutans* in the biofilms. In addition, the thickness of EPS is irrelevant to eDNA aggregation within biofilms.

## Introduction

*Streptococcus mutans* is one of the etiological agents of dental caries, one of the most common human infectious diseases^1–3^. *S. mutans* generates glucosyltransferases (GtfB, GtfC, and GtfD) that produce insoluble and soluble glucans utilizing sucrose, and these glucans enhance the aggregation of oral bacteria and form biofilms^4–6^. The biofilms are encased by extracellular matrix that is composed of exopolysaccharides (EPSs), lipids, proteins, and extracellular DNA (eDNA)^7–9^. The importance of eDNA as one of the major components of biofilms was first shown in *Pseudomonas aeruginosa*^10^. Accordingly, researchers have studied the origin of eDNA, and some researchers suggested that controlled cell death and lysis of bacteria release eDNA, whereas others showed that eDNA was secreted naturally from bacteria^11–13^.

Some researchers have shown eDNA in the form of membrane vesicles, nanofibers, or “yarn-like” or “sweater-like” structures and revealed that eDNA stabilizes biofilms^8,14–17^. Biofilms can act as a barrier to prevent inflow of antibiotics; thus, the bacteria in biofilms can be resistant to antimicrobial agents up to 1,000-fold, which makes eDNA an important target to control biofilm^18^.

Although researchers have studied eDNA of *S. mutans* for more than a decade, most studies focus on the origin and role of eDNA in biofilms, and there is no study on the factors associated with the levels of eDNA within biofilms. Thus, in the present study, we use hydroxyapatite (HA) discs to form biofilms and different concentrations of sugar to investigate any factors that affect the levels of eDNA in *S. mutans* biofilms.

## Results

### The influence of different media on acidogenicity of *S. mutans* biofilms

**Figure 1:**
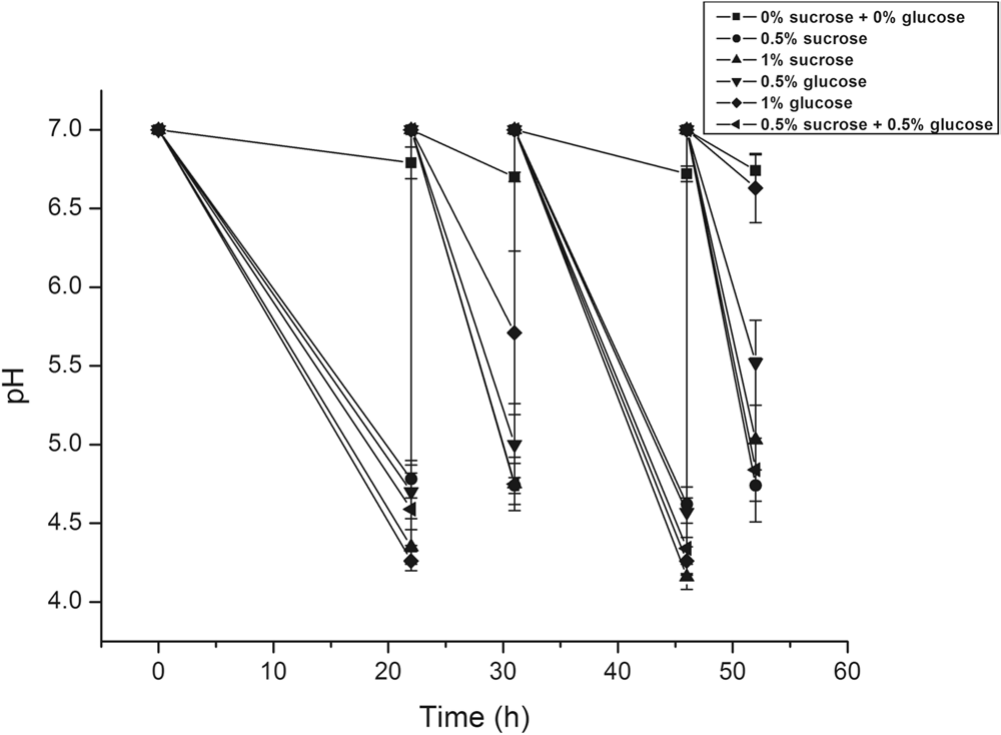
Acidogenicity of media after biofilm formation. pH values of the 6 different media were assessed at 22, 31, 46, and 52 h. Data represent the mean and standard deviation of 6 independent experiments.

The influence of the media (0% sucrose +0% glucose, 0.5% sucrose, 1% sucrose, 0.5% glucose, 1% glucose, and 0.5% sucrose +0.5% glucose) on acidogencity of *S. mutans* biofilms was investigated (Fig. 1). When there was no sucrose and glucose (0% sucrose +0% glucose), pH was around 7 and was maintained (6.73 ± 0.04) during the experiment. *S. mutans* in biofilms had a similar pattern of acidogenicity (4.46 ± 0.21) when the biofilms were in the media containing glucose or sucrose over 15 h after media change (at 22 h and 46 h). The biofilms had various pH values, but were higher in sucrose than glucose when they were in the media less than 9 h after media change (at 31 h and 52 h).

### Viability and EPS of *S. mutans* in biofilms formed in different media

**Figure 2:**
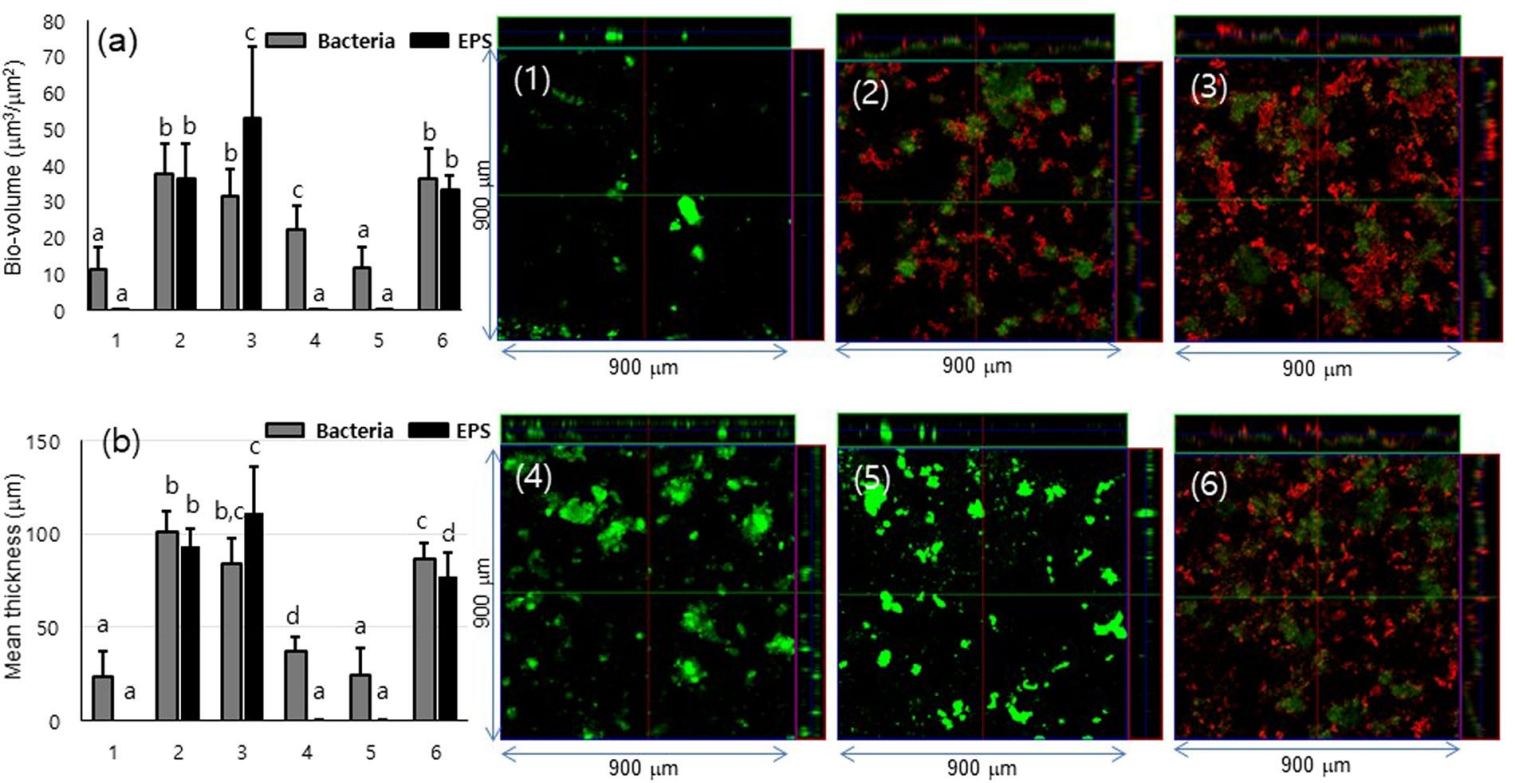
Effects of different media on bio-volume and thickness of bacteria and EPS. The images of the biofilms formed in 6 different media (1. 0% sucrose +0% glucose, 2. 0.5% sucrose, 3. 1% sucrose, 4. 0.5% glucose, 5. 1% glucose, and 6. 0.5% sucrose +0.5% glucose) using confocal laser scanning microscopy, and (**a**) bio-volume and (**b**) thickness of bacteria (green) and EPS (red) of the biofilms were quantified by COMSTAT software. Data represent the mean and standard deviation of 3 independent experiments. Values followed by the same superscript are not significantly different (p > 0.05).

The viability and EPSs of *S. mutans* in the 52-h-old biofilms were analyzed via bio-volume (Fig. 2a) and mean thickness (Fig. 2b) using confocal laser scanning microscopy (CLSM). Bio-volume and mean thickness of bacteria were higher in 0.5% sucrose than 1% sucrose, and the same pattern was shown in glucose, whereas EPSs had lower bio-volume and mean thickness in 0.5% sucrose than 1% sucrose. In 0.5% sucrose +0.5% glucose, bio-volume and mean thickness of bacteria were higher than those in 1% sucrose and lower than those in 0.5% sucrose, and bio-volume and mean thickness of EPS were lower than those in 0.5% and in 1% sucrose. According to the representative bacterial and EPS images from the CLSM study shown in Fig. 2 (1–6), while *S. mutans* produced abundant EPSs in sucrose media, there were almost no EPSs detected in 0% sucrose +0% glucose media or in glucose media. In addition, *S. mutans* showed more EPSs in 0.1% sucrose than in 0.5% sucrose or 0.5% sucrose +0.5% glucose.

### The concentrations of *S. mutans* eDNA within biofilms are cell-number dependent

**Figure 3:**
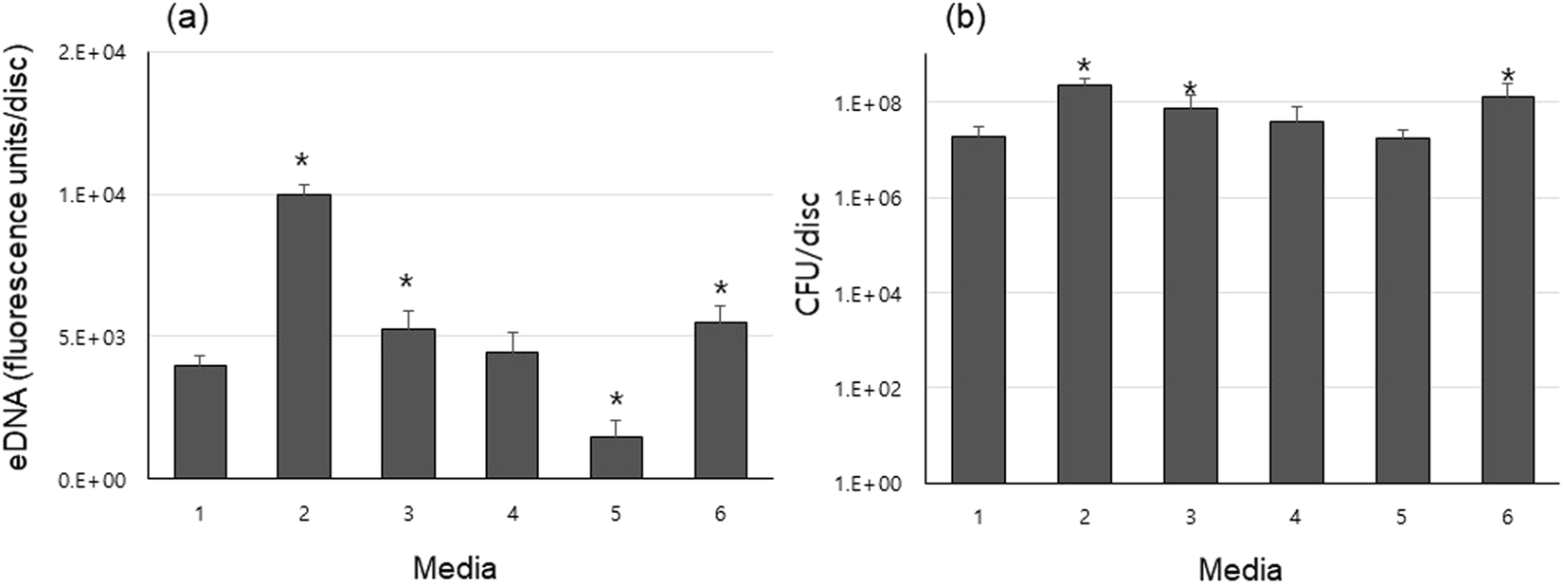
(**a**) eDNA collected from the 52-h-old biofilms cultured in 1. 0% sucrose +0% glucose, 2. 0.5% sucrose, 3. 1% sucrose, 4. 0.5% glucose, 5. 1% glucose, and 6. 0.5% sucrose +0.5% glucose. (**b**) Bacterial viability of 52-h-old *S. mutans* UA 159 biofilms cultured in each media (1–6). Data represent the average and standard deviation of 3 independent experiments. Values were considered statistically significant when the p value was less than 0.05 (p < 0.05) compared to 0% sucrose +0% glucose media.

**Figure 4:**
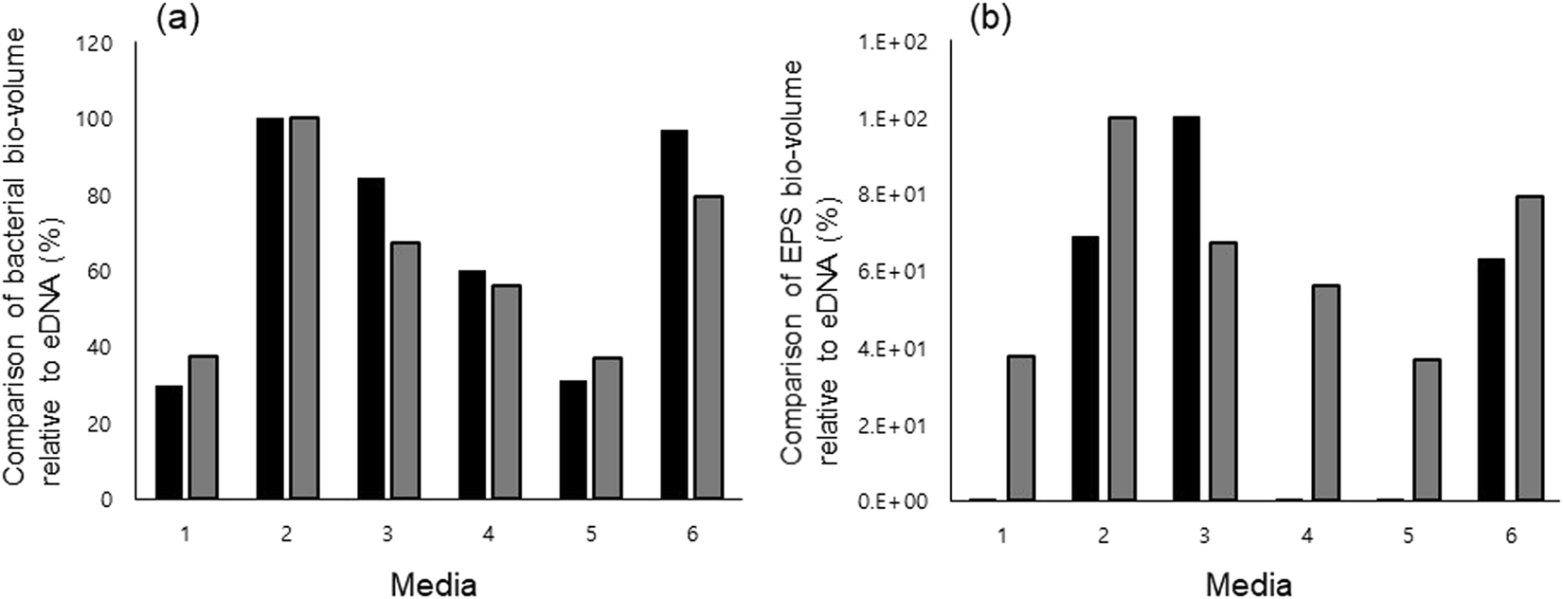
Comparison of bio-volume relative to eDNA. (**a**) Bacteria bio-volume relative to eDNA. Black bar – bio-volume of bacteria, and gray bar – eDNA in biofilm. The highest bar among bacteria bio-volume samples (no. 2) is fixed as 100, and the others are calculated accordingly. Among eDNA samples, no. 2 is fixed as 100. (**b**) EPS bio-volume relative to eDNA. Black bar – bio-volume of EPS, and gray bar – eDNA in biofilm. The highest bar among EPS bio-volume samples (no. 3) is fixed as 100, and the others are calculated accordingly. Among eDNA samples, no. 2 is fixed as 100. 1. 0% sucrose +0% glucose, 2. 0.5% sucrose, 3. 1% sucrose, 4. 0.5% glucose, 5. 1% glucose, and 6. 0.5% sucrose +0.5% glucose.

The levels of *S. mutans* eDNA in the 52-h-old biofilms (Fig. 3a) coincided with the numbers of CFUs in the biofilms (Fig. 3b). The biofilms grown in 0.5% glucose and sucrose had more *S. mutans* than those formed in 1% concentrations, and eDNA levels were approximately 1.8 fold higher in 0.5% sucrose than 1% sucrose and about 3 fold higher in 0.5% glucose than 1% glucose. *S. mutans* in biofilms had more CFUs and eDNA in 0.5% sucrose +0.5% glucose than in 1% sucrose and less than in 0.5% sucrose. To investigate the relationship between eDNA levels in biofilms and the bio-volume of bacteria or EPS in the biofilms, relative eDNA levels were calculated and compared (Fig. 4).

## Discussion

In the present study, we sought to characterize the factors associated with eDNA aggregation in biofilms, and the results of this study show that eDNA levels in biofilms are related to the number of *S. mutans* in the biofilms.

*S. mutans* is an oral bacteria that generates glucosyltansferases (Gtf B, Gtf C, and Gtf D) to produce insoluble and soluble glucans, and these glucans promote the formation of biofilms^19^. The oral bacteria that produce acids in these biofilms are considered to acidify the microenvironment by reducing the pH of plaque, enhancing the growth of acidogenic and aciduric bacteria, and leading to dental caries^20,21^.

According to the results in this study, when acidogenicity of biofilms was detected at 52 h, the pH values were inversely proportional to the number of CFUs of *S. mutans* in the biofilms. Furthermore, the acidogenicity in glucose media that formed no EPS was similar to that in sucrose media after overnight culture. This suggests that acidogenicity of *S. mutans* is closely associated with number of bacteria in the presence of sugar and does not correlate to the thickness of EPS, a major component of biofilms. This needs further investigation.

The viability of *S. mutans* was affected by sugar type. We assumed that the bacterium could have better viability in media that had higher concentrations of sugar since *S. mutans* grows faster and more abundantly in sucrose media than glucose media^22,23^. However, in this study, *S. mutans* grew more in 0.5% sucrose than in 1% sucrose and had less EPS in 0.5% sucrose than 1% sucrose media, suggesting that EPS thickness in biofilms is related to the concentration of sucrose, whereas CFUs do not coincide with sucrose concentration. However, Cai *et al*. showed that the thickness of EPS in biofilms can increase in up to 1% sucrose, while that in greater than 1% sucrose media decreased^24^, suggesting that EPS thickness is also not associated with sucrose concentration greater than 1% in culture media. In glucose media, *S. mutans* also grew more abundantly in 0.5% than 1%. While the biofilms grown in the combination of 0.5% sucrose +0.5% glucose displayed lower CFUs and EPSs than those formed in 0.5% sucrose, they showed higher CFUs and less EPSs than those grown in 1% sucrose. This can be explained as the addition of glucose can reduce the synthesis of EPSs and growth of *S. mutans*^25,26^.

Some researchers suggested that *S. mutans* produce more eDNA in biofilms than in planktonic culture^15^. However, as shown in 0.5% sucrose, when there were more bacteria in biofilm, the eDNA levels were higher than in 1% sucrose and 0.5% sucrose +0.5% glucose. Interestingly, *S. mutans* grown on discs in glucose media showed the same pattern, which suggests that eDNA levels of *S. mutans* are related to the number of bacteria regardless of EPS. The result also showed that *S. mutans* in biofilms with EPS has more eDNA, as glucans produced by *S. mutans* in sucrose media provide binding sites to bacteria, allowing *S. mutans* to aggregate more in biofilms^27,28^. This can be explained by a study that showed the exposure of *S. mutans* to the nutrient flow was lower when sucrose concentration in culture media was higher and accordingly, adhesion of *S. mutans* increases as sucrose levels increase up to a certain concentration (0.45%) and then it decreases as sucrose concentration increases. However, water-insoluble EPSs and dry-weight increase in a sucrose-level dependent manner in biofilms formed in the media less than 1% sucrose^24^.

Regarding the single-species biofilms used in this study, this phenomenon could be different in multi-species biofilms. According to Castillo Pedraza *et al*., UA159 had more biomass and more eDNA in single-species biofilms than multi-species biofilms^29^. Micro-environmental conditions and amounts of carbohydrates also affect EPS and eDNA contents in multi-species biofilms^30,31^.

In summary, the results of this study provide new findings that eDNA levels in biofilms are dependent on the CFUs in the biofilms, that acidogenicity of *S. mutans* biofilms is related to the CFUs in the biofilms, and eDNA levels in biofilms are not related to the thickness of EPS of the biofilms.

## Materials and Methods

### Bacterial strain, culture conditions, and biofilm preparation

*S. mutans* UA159 was cultured in ultrafiltered (10-kDamolecular-mass-cutoff membrane) tryptone-yeast extract broth (UFTYE, pH 7.0) containing 1% (wt/vol) glucose aerobically at 37 °C under 5% CO_2_ without agitation. For biofilm formation, hydroxyl-apatite (HA) discs (2.93 cm^2^; Clarkson Chromatography Products, Inc. South William sport, PA. USA) placed in vertical in 24-well plates in UFTYE with 0% sucrose +0% glucose, 0.5% sucrose, 1% sucrose, 0.5% glucose, 1% glucose, or 0.5% sucrose +0.5% glucose and overnight-cultured *S. mutans* UA159 (5 × 10^6^ colony-forming unit (CFU)/ml)^32^. The biofilms were grown undisturbed for 22 h and the media was changed at 22, 31, and 46 h. Brain heart infusion (BHI; Difco, Detroit, MI, USA) agar plates were prepared with an addition of 1.5% (wt/vol) of agar (Difco Laboratories, Detroit, MI, USA).

### Measurement of pH, eDNA, and CFU count

The pH values of biofilms were determined at 22, 31, 46, and 52 h by measuring the old culture media when the media was changed to investigate changes in *S. mutans* biofilm acidogenicity in the presence of 0% sucrose +0% glucose, 0.5% sucrose, 1% sucrose, 0.5% glucose, 1% glucose, or 0.5% sucrose +0.5% glucose. eDNA of *S. mutans* was measured as previously described with some modifications^15,33^. Briefly, *S. mutans* UA159 was cultured overnight in UFTYE supplemented with 1% glucose. On the next day, approximately 6 × 10^5^ CFU/ml bacteria was transferred into 24-well plates containing HA discs and UFTYE with 0% sucrose +0% glucose, 0.5% sucrose, 1% sucrose, 0.5% glucose, 1% glucose, or 0.5% sucrose +0.5% glucose and cultured for 52 h. The bacteria on the discs were washed twice with 0.89% NaCl and collected in 0.89% NaCl by scraping them from the discs using a spatula. The bacteria collected from each disc was centrifuged at 10,000 × g at 4 °C for 10 min, and the pellet was re-suspended in 0.89% NaCl and sonicated (10 s, twice at 20% energy level on ice with 1 min interval) to break the biofilms and separate eDNA from the surface of bacteria using a sonifier (VCX 130PB; Sonics and Materials Inc., Newtown, CT, USA). An aliquot (0.1 ml) of the sonicated pellet was serially diluted and plated on BHI agar plates to count the CFUs of the bacteria. The rest were centrifuged at 10,000 × g at 4 °C for 10 min. The supernatant was filtered using 0.22-μm syringe filters (Millipore, USA) to gain pellet-free supernatant. For eDNA measurement, the cell-free and pellet-free supernatants were treated with DNA-binding dye, SYBR Green I (Invitrogen). The samples were excited at 485 nm and emitted at 535 nm. The eDNA concentration was measured using a fluorescence microplate reader (HIDEX). Four independent experiments were performed to measure eDNA and count CFUs.

### Confocal laser scanning microscopy

CLSM was performed to determine the changes in *S. mutans* biofilm formation. One μM of Alexa Fluor 647-labeled dextran conjugate (10,000 MW; absorbance/fluorescence emission maxima 647/668 nm; Molecular Probes Inc., Eugene, OR, USA) was added to 0% sucrose +0% glucose, 0.5% sucrose, 1% sucrose, 0.5% glucose, 1% glucose, or 0.5% sucrose +0.5% glucose BHI broth with *S. mutans* UA159 (6 × 10^5^ CFU/ml) at 0, 22, 31, and 46 h. The fluorescence-labeled dextran serves as a primer for GTFs and can be simultaneously incorporated during the extracellular polysaccharide matrix synthesis over the course of the biofilm development. After 52 h, the bacterial cells in the biofilms were labeled by incubation with 2.5 μM SYTO 9 green fluorescent nucleic acid stain (480/500 nm; Molecular Probes Inc.) for 30 min. CLSM imaging of the biofilms was performed using an LSM 510 META (Carl Zeiss, Jena, Germany) microscope equipped with argon ion and helium-neon lasers. Four independent experiments were performed, and five image stacks per experiment were collected (n = 20). The bio-volume (μm^3^/μm^2^) and mean thickness (μm) of bacterial micro-colonies and EPSs were quantified from the confocal stacks using COMSTAT.

### Evaluation of eDNA production according to bacteria bio-volume or EPS bio-volume

To compare the eDNA levels in biofilms to bacteria bio-volume or EPS bio-volume of the biofilms, the relative ratio of eDNA level in each biofilm was calculated as follows: among the eDNA levels in biofilms formed in 6 different media, the highest was fixed as 100, and the rest were calculated accordingly. Relative ratios of bacteria bio-volume and EPS bio-volume were calculated in the same way.

$${\rm{Relative}}\,{\rm{ratio}}\,{\rm{of}}\,{\rm{eDNA}}\,{\rm{level}}=\frac{{\rm{eDNA}}\,{\rm{in}}\,{\rm{each}}\,{\rm{biofilm}}}{{\rm{The}}\,{\rm{highest}}\,{\rm{eDNA}}}\times {\rm{100}}$$

### Statistical analysis

All experiments were performed at least three times. The intergroup differences were estimated by one-way analysis of variance (ANOVA). The data are presented as mean ± standard deviation (SD). A p value was considered statistically significant when it was less than 0.05 (p > 0.05).

## Data Availability

The data generated and analyzed in this study are available from the corresponding author upon request.
